# Sterol and lipid metabolism in bees

**DOI:** 10.1007/s11306-023-02039-1

**Published:** 2023-08-29

**Authors:** Samuel Furse, Hauke Koch, Geraldine A. Wright, Philip C. Stevenson

**Affiliations:** 1 Royal Botanic Gardens, Kew Green, Kew, Surrey, TW9 3AB UK; 2 Department of Biology, University of Oxford, Oxford, OX1 3SZ UK; 3 Natural Resources Institute, University of Greenwich, Chatham, Kent, ME4 4TB UK

**Keywords:** *Apis mellifera*, *Bombus terrestris*, Lipids, Sterols, Fatty acids, Nutrition, Queen bee acid, Phospholipids, Triglycerides

## Abstract

**Background:**

Bees provide essential pollination services for many food crops and are critical in supporting wild plant diversity. However, the dietary landscape of pollen food sources for social and solitary bees has changed because of agricultural intensification and habitat loss. For this reason, understanding the basic nutrient metabolism and meeting the nutritional needs of bees is becoming an urgent requirement for agriculture and conservation. We know that pollen is the principal source of dietary fat and sterols for pollinators, but a precise understanding of what the essential nutrients are and how much is needed is not yet clear. Sterols are key for producing the hormones that control development and may be present in cell membranes, where fatty-acid-containing species are important structural and signalling molecules (phospholipids) or to supply, store and distribute energy (glycerides).

**Aim of the review:**

In this critical review, we examine the current general understanding of sterol and lipid metabolism of social and solitary bees from a variety of literature sources and discuss implications for bee health.

**Key scientific concepts of review:**

We found that while eusocial bees are resilient to some dietary variation in sterol supply the scope for this is limited. The evidence of both *de novo* lipogenesis and a dietary need for particular fatty acids (FAs) shows that FA metabolism in insects is analogous to mammals but with distinct features. Bees rely on their dietary intake for essential sterols and lipids in a way that is dependent upon pollen availability.

## Introduction

Insects that visit flowers often transfer pollen, the male gamete, from the anther of one flower to the stigma of a conspecific flower. This process facilitates plant reproduction in around 90% of angiosperms and 75 of the 100 most important crops, accounting for 35% of global crop yields (Klein et al., 2007; Ollerton et al., 2011). Several anthropogenic drivers, including climate change, agricultural intensification, habitat loss and disease have led to declines in pollinator abundance and diversity. It has also restricted nutrient provision from floral resources which could have consequences for pollinator health (Baude et al., 2016; Brodschneider & Crailsheim, 2010; Potts et al., 2016). It is likely that the combination of these effects has increased their magnitude (Vanbergen & Initiative, 2013).

Large-scale changes in plant distribution and diversity have consequences for nutrient availability for virtually all wild animals and some domesticated ones. This is especially important for insect pollinators including social and solitary bees hereafter referred simply as bees. A fundamental understanding of their nutritional requirements and of the nature of their nutritional resources would help us characterise the link between large-scale changes in ecosystems and the survival and health of pollinators from individuals to communities (Stevenson et al., 2022). It would also help us to develop mitigation strategies that could improve availability and quality of nutritional resources. For example, compounds such as phytosterols are essential metabolites for bees but their availability in pollen varies dramatically between plant taxa (Zu et al., 2021). Not all sterols found in pollen are suitable for all honey bees or solitary bees (Svoboda et al., 1980; Vanderplanck et al., 2020) but the consequences for the development of pollinators (growth from egg to maturity) and challenges to metabolic health caused by a limited range of pollen sources, and thus limited range of nutrients in the landscape, is unknown. Understanding how individual nutrients are acquired through floral pollen by insect pollinators would tell us not only about how floral diversity influences pollinator health but also what the effects on bees are of changes in primary pollen resources. Such knowledge would make it possible to model, and therefore predict, how environmental change affects impacts pollinator biodiversity.

**Fig. 1:**
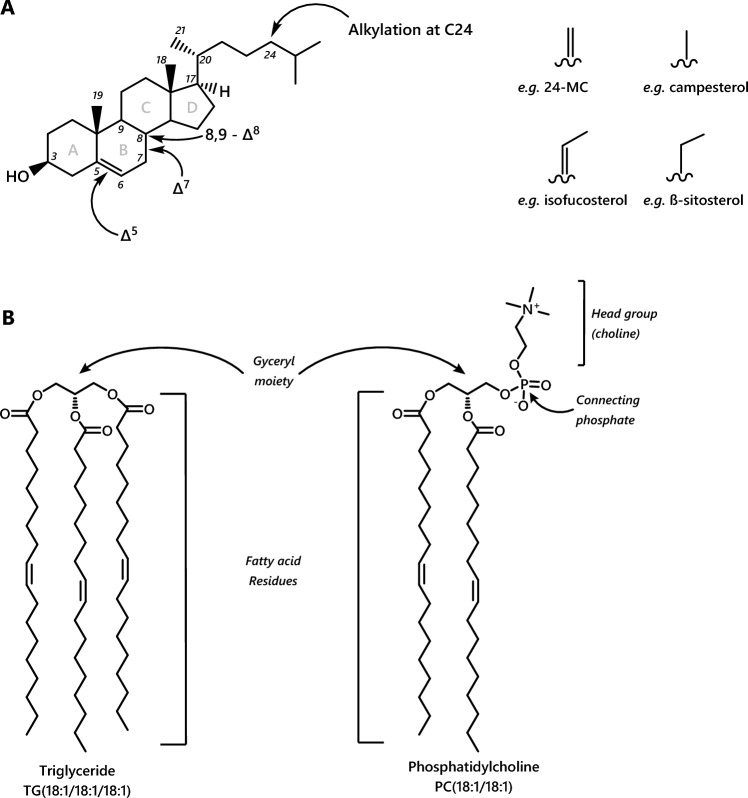
A structural guide to different lipids. **A**, phytosterols; these molecules typically differ by the position of the double bond in the A ring and alkylation at the C24 position. However, other elaborations have been observed;** B**, Triglyceride (left) and phosphatidylcholine (right). NB. Replacement of the choline moiety by other moieties gives rise to other phospholipid classes such as phosphatidylethanolamines and phosphatidylinositols. These are glyceryl lipids as both comprise glycerol

Pollen is the principal dietary source of sterols, essential FAs, triglycerides, phospholipids, with collection of oils also observed in some solitary bees (Neff & Simpson, 2017). A guide to the structures of sterols, triglycerides and phospholipids is shown in Fig. 1. These compounds have a variety of possible functions and destinations in vivo, with many probably as yet undiscovered. This frustrates efforts to define the role of individual lipids. However, generally, phospholipids are membrane components whereas triglycerides are a way of storing and distributing energy. Sterols are membrane components and in bees, and a starting material for producing steroid hormones. Fatty acids, components of both triglycerides and phospholipids, can be hydroxylated to produce signalling molecules. Molecular studies show that all of these compounds are found in pollen, with the precise composition varying considerably between plant species (Ischebeck, 2016; Zu et al., 2021). Metabolic studies in mammals have shown that a shortage of essential dietary nutrients can lead to poor health and even death, as can an excess of such nutrients. Excess nutrition is the principle underlying cause of the obesity crisis in humans and in companion animals. More complicated metabolic phenotypes have also been recorded (Cropley et al., 2016; Dearden et al., 2018; Lawson, 2013; McIntyre et al., 2019; Stephensen, 1999; Tarry-Adkins & Ozanne, 2017; Wei et al., 2003), including the ‘double burden’ where some nutrients are in excess and others are lacking. Nutritional programming of offspring by the metabolic phenotype of their parents (Blackmore et al., 2014; Fernandez-Twinn et al., 2017; Fernandez-Twinn et al., 2019; Lawson, 2013; Loche et al., 2018; Musial et al., 2017) and dietary intake (Furse et al., 2021; Furse et al., 2022c; Morgan et al., 2020; Watkins et al. 2018; Watkins & Sinclair, 2014; Watkins et al., 2017) is gaining research attention. In mammals, both the parents’ metabolic phenotype and nutrient intake shape the development and metabolism of offspring at least two generations hence (Furse et al., 2021; Furse et al., 2022c). Such detailed studies have rarely been conducted in pollinator insect species such as bees, however. Here, we critically review the existing knowledge of lipid composition and metabolism of bees and bee tissues in the broadest sense, including phospholipids, triglycerides, fatty acids and sterols, and identify knowledge gaps. This is important because it can inform nutrient interventions from food supplements to floral diversity to improve bee health.

## Sterols

Sterols are organic biomolecules that are derived from squalene. The sterol metabolism of insects has received significant attention in the last half-century (Nes, 2011; Svoboda & Feldlaufer, 1991). An important step in understanding sterol metabolism in biological systems relies upon the characterisation of their sterol composition (sterolome). Traditionally, sterols have been identified chromatographically (Svoboda et al., 1983b; Svoboda & Lusby, 1986; Svoboda et al., 1980), with gas chromatography-mass spectrometry used more recently (Lusby et al., 1993; Pilorget et al., 2010; Takatsuto & Omote, 1989; Zu et al., 2021). Methods for profiling the sterol composition using liquid chromatography-mass spectrometry have been reported (Honda et al., 2010) as has comprehensive natural product discovery and structural determination using a combination of mass spectrometry and NMR (Kodai et al., 2007). Comprehensive sample prep methods for samples or data collection for medium- and high throughput sterol profiling in insects have not yet been reported and would benefit this field of study. These techniques have also focused on profiling hexane-soluble sterols, with less work on the hydroxylated sterol derivatives such as steroid hormones. This contrasts with human studies, where steroid profiling is well developed (Boggs et al., 2016; Schiffer et al., 2019; Yuan et al. 2020).

Sterolomics in the context of sterol metabolism in bees requires analyses of sterols from a variety of samples, including pollen and the bees themselves. There are reports of at least two steroid hormones in bees, with some work on their metabolism (Yamazaki et al., 2011) however none yet of bee bread, pollen or royal jelly which would inform how the bee diet influences the sterolome of the bees that consumes them, limiting scope for comparing, e.g. wild and laboratory-grown bees as well as solitary and eusocial ones. The work done to date has therefore provided fundamental information about phytosterols in the dietary intake of bees and which sterols are found where, with only formative studies of steroid metabolism. This work has therefore shown the range of phytosterols in bees, discussed first.

The sterol profile (sterolome) of eusocial bees is remarkable in that adults with very different sterolomes can live for long periods under both laboratory and wild conditions. This variation is matched by differences between individuals and between castes (Vanderplanck et al., 2020). Collections of adult honey bees from field sites where pollen from one species formed the majority of the dietary intake showed that bees can survive when the sterol fraction of the pollen was dominated by either 24-methylenecholesterol, ß-sitosterol or isofucosterol (Svoboda et al., 1983b), though it is expected that new pollen was stored whilst other brood provision was used, meaning the stored pollen could have modulated the sterol profile of the new pollen. A guide to sterol structure is shown in Fig. 1A. Some wild bees have sterol profiles that resemble those of the pollen they collect (Behmer, David Nes 2003; Vanderplanck et al., 2020) or accumulate 24-methylenecholesterol (Vanderplanck et al., 2020). Laboratory studies of honey bees show that the sterolome of bees is influenced by their diet (Barraud et al., 2022; Takatsuto et al., 1989).

This diversity is partly due to the variety of sterols provided in nature. There are at least 25 sterols recorded from plant pollen (Zu et al., 2021), with as many as twelve sterols detected in pollen of any individual species (Rasmont et al., 2005; Svoboda et al., 1983b; Vanderplanck et al., 2020; Zu et al., 2021). The pollen sterolome of *Populus fremontii* and *Hypochoeris radicata* is dominated by cholesterol (C_27_, δ5; Standifer et al., 1968; Vanderplanck et al., 2020), whereas the sterolome of *Zea mays, Prunus avium* and *P. spinosa* is dominated by 24-methylenecholesterol (C_28_, δ5; Guo et al., 1995; Standifer et al., 1968; Villette et al., 2015; Zu et al., 2021). The pollen of *Alnus glutinosa* and *Calluna vulgaris* is mainly ß-sitosterol (C_29_, δ5; Standifer et al., 1968; Vanderplanck et al., 2020), whereas that of *Helianthus* spp. is mainly avenasterol (C_29_, δ7; Takatsuto & Omote, 1989) (For structural guide, see Fig. 1A). Bees can also acquire sterols from at least one non-pollen source; ergosterol has been found to come from the fungal ascomycete *Zygosaccharomyces* (Paludo et al., 2018). These profiling studies show that a greater number of phytosterols are available to and used by bees and that this varies by taxa (Vanderplanck et al., 2018; Vanderplanck et al., 2020).

The second reason why bees have a relatively varied sterolome is that they carry out no bulk onward metabolism of sterols (Svoboda & Feldlaufer, 1991; Svoboda et al., 1982). They are not able to dealkylate sterols, i.e. remove the methyl or ethyl group at C24 (Fig. 1A) and thus do not turn campesterol or ß-sitosterol into cholesterol (Svoboda et al., 1981). This preserves the C28 and C29 sterols taken up in the gut (24MC, isofucosterol, etc.). The only onward metabolism of sterols in bees is the use of small amounts of cholesterol and campesterol to make steroid hormones, however this represents only a small amount of the total (< 0·1%).

Although adult bees show a remarkable variety of sterol profiles, the amount of viable offspring differs markedly between sterol intakes in both *Apis* and *Bombus* species (Vanderplanck et al., 2018). This implies that sterol intake is critical for successful development. Laboratory feeding experiments of honey bees have shown that diets enriched in a known sterol were associated with a higher abundance of that sterol in their prepupae. However, 24-methylenecholesterol remained the most abundant sterol in the bees in all cases, up to 71% (Svoboda et al., 1980) suggesting selective accumulation. Importantly, only the prepupae supplemented with 24-methylenecholesterol survived to 12w, all other feeding groups including the control died out by 8w. In the 24-methylenecholesterol-supplemented group, the abundance of the supplemented sterol was around 70% by 8w and remained at that level. This evidence shows that 24-methylenecholesterol is essential to honey bees, however it is unclear whether others bulk sterols are too. Studies of *Bombus terrestris* indicate that this species probably has similar requirements, for example that brood production correlates with 24-methylenecholesterol availability (Vanderplanck et al., 2018; Vanderplanck et al., 2014), but specific feeding studies where purified sterols were fed to broodstock have not yet been reported. This therefore suggests that both species have a similar reliance on 24-methylenecholesterol. Therefore, along with the starting materials used to make steroid hormones, there are at least three sterols that are essential for bees: 24-methylenecholesterol, campesterol and cholesterol. However, C_29_ sterols isofucosterol and ß-sitosterol are found in Royal Jelly, suggesting they also have a role. It is not known whether this role is essential or not. This variation and reliance on several sterols naturally raises questions about how this profile is controlled.

Several studies provide evidence for how the sterolome is managed in bees. First, honey bees do not excrete more campesterol, ß-sitosterol and isofucosterol (C24-alkylated sterols) than 24-methylenecholesterol, suggesting that uptake is not specific (Feldlaufer & Harrison, 2020). Second, the sterol composition of prepupae, Royal Jelly (Kodai et al., 2007), queen bee ovaries (Barbier, 1987) and of hypopharyngeal glands (Svoboda et al., 1986) are consistent with intake from pollen. Third, although there is evidence for concentration of sterols in the hypopharyngeal gland, there is no evidence for storage of sterols in the way that triglycerides are stored in the fatbody. This suggests that dietary availability of suitable sterols has always been sufficient.

The reliance on dietary intake for 24-methylencholesterol means it is not surprising that it is typically the most abundant sterol in honey bee-collected pollen (Zu et al., 2021). This indicates that pollen selection is the most important factor in sterol acquisition as with other essential nutrients such as protein and polyunsaturated fatty acids. Bees may be able to detect fats with antennae (Muth et al., 2018) however there is no evidence that this is possible with sterols. A comprehensive survey of stored pollen may reveal how consistent bees are across ecosystems, however this has not been reported. Taken together, this evidence suggests first that dietary intake is key to sterol intake however the apparent weaknesses in control suggest that the selection pressures bees have been exposed to have not included sterol famines. However, one sterol dominates the sterolome *in apia*, 24-methylenecholesterol.

**Fig. 2:**
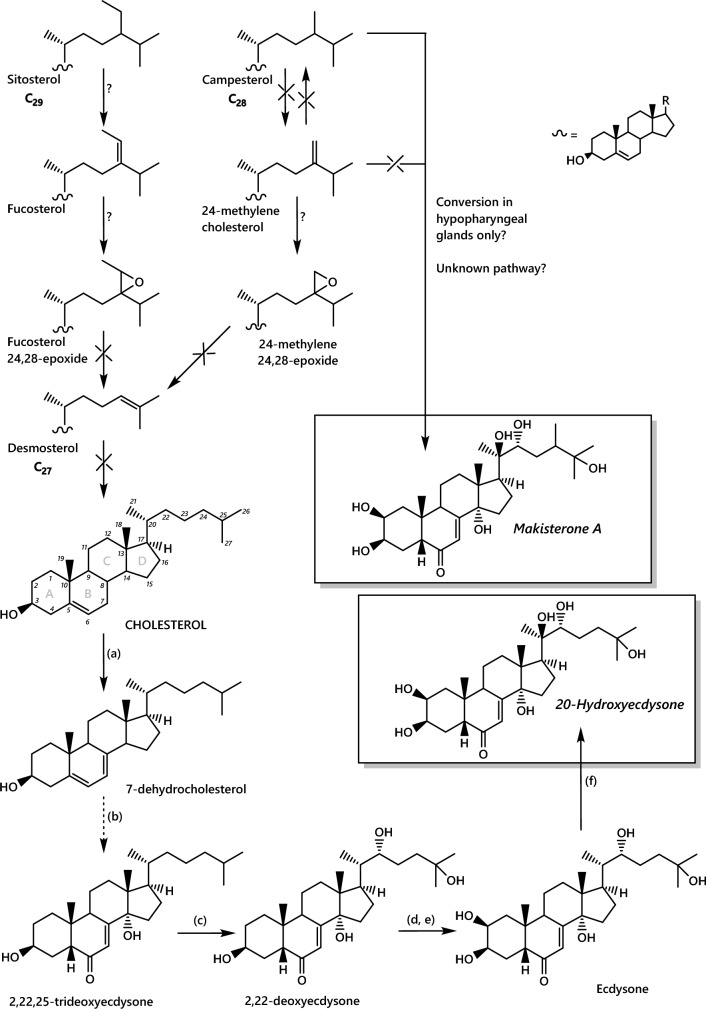
Current understanding of sterol and steroid hormone metabolism in adult *Apis mellifera*. Known enzymes/conversions: (a) Neverland 7,8-dehydrogenase; (b) uncharacterised steps that include CYP307 and NM-G/SRO; (c) CYP306A1 25-hydroxylase; (d) CYP302A1 22-hydroxylase; (e) CYP315A1 2-hydroxylase; (f) CYP314A1 20-hydroxylase. The arrows for conversions known not to be possible are crossed. Unknown conversions are labelled with a question mark. Data from original studies (Feldlaufer et al., 1986b; Svoboda et al., 1981, 1983a; Yamazaki et al., 2011). Carbon numbers and ring nomenclature for cholesterol shown for reference

The evidence for both a high abundance of 24-methylenecholesterol in honey bees and it not being metabolised points firmly towards one or more physical roles. It is remarkable that it is not used in the biosynthesis of steroid hormones as it is structurally very similar to campesterol, that is (Fig. 2). One structural role for 24-methylenecholesterol for which there is clear evidence is in the royal jelly protein 1 oligomer. 24-Methylenecholesterol lends the protein hydrophobicity and provides a possible membrane anchor (Tian et al., 2018). However, this does not explain the dominance of 24-methylenecholesterol in the bee sterolome. The role we would expect is as a plasma- and endoplasmic reticulum membrane component, analogous to cholesterol in mammals. However, this has not been tested specifically. The physical behaviour of membranes differs generally according to the sterol(s) present (Clarke et al., 2006; Hsueh et al., 2005; (McConnell & Radhakrishnan, 2006; Orädd et al., 2009) and specifically, structural differences in the alkyl chain section of sterols modulate the phase behaviour of sterols in model membranes. Campesterol and brassicasterol reduce the melting temperature of model lipid membranes more than cholesterol (Benesch & McElhaney, 2014), indicating that sterols with a C24 alkyl group have a fluidising effect on membranes. This suggests that the presence of 24-methylenecholesterol in bees’ membranes means they may be more resistant to colder temperatures than if cholesterol dominated or need different fatty acids for their membrane phospholipids. If the longer alkyl branch chain in ß-sitosterol (ethyl group at C24) has a yet more fluidising effect than that of campesterol (methyl group at C24), the physical behaviour of sterols might provide an explanation for field observations of *Bombus terrestris* foraging almost exclusively on strawberry tree (*Arbutus unedo*) pollen during autumn in the Mediterranean. The pollen from *A. unedo* is low in 24-methylenecholesterol but high in ß-sitosterol and is collected during a cooler season (Rasmont et al., 2005).

The physical role for sterols contrasts sharply with their biochemical role as the starting materials for steroid synthesis. Svoboda and colleagues in the 1980s developed the first detailed understanding of steroid biosynthesis in honey bees review (Svoboda & Feldlaufer, 1991). These studies showed that honey bees can make the moulting hormones 20-hydroxy ecdysone and makisterone A from cholesterol and campesterol respectively (Feldlaufer et al., 1986a; Yamazaki et al., 2011), (Fig. 2). However, like other insects they are unable to biosynthesise cholesterol (Behmer, David Nes 2003; Clark & Bloch, 1959), demonstrated by sterol synthesis inhibitors having little effect on the abundance of sterols or on larval development (Svoboda et al., 1987). It is expected that bees have specific uptake of cholesterol, as mammals and *Drosophila* do NPC1 (Dixit et al., 2007; Jing & Behmer, 2020). It is not clear whether there is specific uptake of campesterol in bees, despite it being present continuously in bees. Furthermore, there is no evidence that bees can dealkylate phytosterols (Svoboda et al., 1983a; Svoboda et al., 1981), preserving both campesterol and 24-methylenecholesterol. Evidence for hormone synthesis from cholesterol however is strong. A study reported by Yamakai et al. (2011) showed that cholesterol is converted to 2-hydroxyecdysone in the brain, fat body and ovary, but not the hypopharyngeal glands in vivo. By contrast, recently Corby-Harris et al. found that makisterone A (derived from campesterol and thus possesses a methyl group on C24, in the alkyl chain, but which is absent from 2-hydroxyecdysone) accelerates autophagy of hypopharyngeal glands (Corby-Harris et al., 2019). This follows work showing that makisterone A is found in queen bee ovaries (Feldlaufer et al., 1986b). The pathways are summarised in Fig. 2. Further work is required to establish the precisely how steroid hormone biosynthesis is controlled. Currently, it is unclear why both ecdysone and makisterone A are required in bees as they are structurally very similar. The presence of two very similar steroid hormones also raises questions about how their biosynthesis is controlled. The simplest means would be for the hormones to be synthesised in different tissues.

The evidence to date on sterol metabolism in bees suggests there is no biosynthesis, a heavy reliance on dietary intake, accumulation for secretion but no internal storage, selective transfer between adults and larvae but careful biosynthesis of steroid hormones. 24-Methylenecholesterol, campesterol and cholesterol all appear to be essential. This raises the important question of how bees meet their need for sterols as the variety in pollen is so wide. Furthermore, the reliance of bees on pollen as their source of sterols implies that sterol deficiency in a landscape could explain loss of diversity of some bee species with specialists being lost first (Biesmeijer et al., 2006). This suggests that the composition of sterols (ratio and total abundance) in floral pollen is important for ensuring the nutritional suitability of a landscape for pollinators. Experiments with directed dietary sterol composition are necessary to complete our understanding of the specificity of sterol needs in bees.

### Phospholipids

**Fig. 3:**
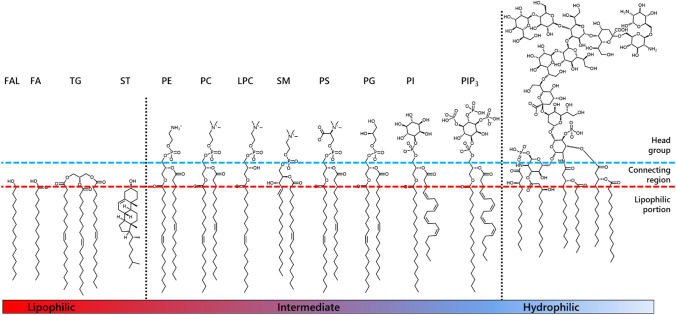
Examples of lipids and their amphiphilicity, adapted from Furse et al. (Furse et al. 2015a). Reading left to right: FAL (fatty alcohol), hexadecanol/cetyl alcohol; FA (fatty acid), FA(16:0)/palmitic acid; TG (triglyceride), TG(18:1/18:1/18:1); ST (sterol), ST(27:1)/cholesterol; PE (phosphatidylethanolamine), PE(18:1/18:1); PC (phosphatidylcholine), PC(18:1/18:1); LPC, (*lyso*-phosphatidycholine), LPC(18:1); SM (sphingomyelin), SM(d18:1/18:1); PS (phosphatidylserine), PS(18:1/18:1); PG (phosphatidylglycerol), PG(18:1/18:1); PI (phosphatidylinositol), PI(18:1/18:1); PIP_3_, phosphatidylinositol-3,4,5-tris-phosphate. A generic lipopolysaccharide is shown on the far right. The horizontal blue and red dashed lines show an approximate delineation between the head group region, the connecting region, and the hydrophobic fraction. The black dotted lines show an approximate delineation between lipophilic and intermediate (left) and intermediate and hydrophilic (right) lipid species

Biological membranes, also known as lipid bilayers, are essential structural features of all cells. Membranes form both the boundary of the cell (plasma membrane) and of cellular compartments. In eukaryotes and prokaryotes phospholipids are essential components of these membranes. The formation of lipid bilayers is energetically favoured and occurs spontaneously on contact of amphiphilic molecules (lipids) with water. This is known as the hydrophobic effect. Lipids from mammalian and bacterial systems have been studied in detail, and examples of different lipid classes are shown in Fig. 3. To date, less work has been done on lipid composition and lipid metabolism in insects. This is partly due to experimental barriers related to animal size; individual *Drosophila* are typically too small to provide enough lipidic material for a single whole-body sample, let alone of individual tissues. However, individual *Apis* spp. and *Bombus* spp. bees are both large enough to provide sufficient material for lipidomics studies at tissue level.

The phospholipid composition of bees has been reported in a few studies in which they were fed either mustard pollen (Singh & Singh, 1996) or pollen collected locally by the bees (Martin et al., 2019; Martin et al., 2022; Wegener et al., 2018). It is not clear what the phospholipid composition of pollen is at present, nor how consistent bees are in their pollen collection with respect to phospholipids, though several studies have been done on fatty acids (*vide infra*). Investigations of lipid composition of bees across developmental stages have been reported (Martin et al., 2019; Martin et al., 2022), as have explorations of individual tissue types such as sperm (Wegener et al., 2013). These studies show that the major phospholipid head groups found consistently in mammals (van Meer, de Kroon 2011) are also present in bees (Hu et al., 2021; Martin et al., 2019; Martin et al., 2022; Singh & Singh, 1996; Wegener et al., 2018; Wegener et al., 2013). There are also apparent tissue lipid composition similarities between bee sperm and human (Furse et al., 2022a), with a high abundance of sphingolyelin and phosphatidylcholine and little of other lipid classes (Wegener et al., 2013). Recent reports on the honey bee lipidome have found that the abundances of the lipid head groups differ between castes (Martin et al., 2019; Singh & Singh, 1996), with phosphatidylcholine (PC) or phosphatidylinositol (PI) reported as being the most abundant in all castes (Wegener et al., 2018). That PI should dominate, or even be the second most abundant lipid, is remarkable in the light of other systems such as drosophila (Guan et al., 2013), algae (Vítová et al., 2021), mice (Furse et al., 2020) and even Gram-positive bacteria (Mastronicolis et al., 2010), in which the total abundance of PI remains below 10%. Several other well-known lipids have been detected, including *lyso-*phosphatidylcholine (LPC), phosphatidylethanolamine (PE), phosphatidylserine (PS), and sphingomyelins (SM) (see Fig. 3 for molecular diagrams). The lipid classes found in bees are broadly consistent with the lipid classes found in other insects such as drosophila (Carvalho et al., 2012), house cricket (Gutiérrez et al., 2022) and bloodworm (Trenti et al., 2022). This suggests that the structure of phospholipid metabolism in bees may be similar to that in mammals, however the details of phospholipid metabolism have not been explored fully insects.

In the studies reported to date, the fatty acid composition of the phospholipids identified contains mainly oleic acid, FA(18:1), with smaller amounts of FA(18:0, 18:2, 18:3) and FA(16:0) (Martin et al., 2019; Martin et al., 2022; Wegener et al., 2018). In a detailed examination of PC in worker bees, there was very little evidence of any other FA (Wegener et al., 2018). This is remarkable because, with evidence about the abundance of lipid classes (Martin et al., 2019), it suggests that the phospholipid profile of bees is centred more on PC or PI than in either mammals or plants, and that the FA profile of each head group (number of configurations within any one lipid class) is narrower than many other species. This has consequences for both the physical behaviour of membranes in bees and other lipid roles. The lipid profiling reported to date suggests that the inositide signals used in bees are not based on PI(18:0/20:4) as they are in mammals, despite similar inositide pathway genes (Wang et al., 2009) and signalling roles for the inositides (Münch & Amdam, 2010). This raises questions about the roles of lipids *in apia* and how lipid activity is sensed and controlled. The roles of induvial isoforms of major lipids have not been investigated specifically in detail in bees to date, however there is some evidence for special roles for lipids comprising polyunsaturated FAs (PUFAs), discussed next.

Dietary intake of linoleic and α-linolenic acids, FA(18:2) and FA(18:3), has been found to be important in cognitive development in bees (Arien et al., 2018; Arien et al., 2015). These FAs are therefore important not only because they support new brood (Arien et al., 2015) but also because they have a functional role in bee development not related to energy provision. This suggests that these polyunsaturated FAs are essential to bees in the way they are to mammals. The role of linoleic and linolenic acids in the development of cognition points towards them having an analogous role in the bee CNS to that of FA(22:6) (docosahexaenoic acid) in the mammalian CNS (Crawford et al., 2014; Dyall, 2015; Furse et al., 2022b; Taha et al., 2016). In mammals, FA(22:6) is essential in grey and white matter as a fatty acid residue in three lipid classes found there (Blusztajn et al., 1979; Farooqui et al., 2000; Vos et al., 1997). Studies of the intake of FA(18:2) and FA(18:3) intake in bees showed that the ratio of these two fatty acids (one of which is ω-6 and the other ω-3) is important for development of associative learning, and that the ratio is more important than the total amount (Arien et al., 2018; Arien et al., 2015). This is consistent with a feeding study that showed that adding FA(18:2) to *Eucalyptus* pollen lowered the lifespan of honey bees by around 50% (Manning et al., 2007). In the same study, the lifespan of honey bees was reduced yet further by supplementation with oleic acid, FA(18:1). However, the mechanism that drives the cognition or longevity effects is not clear. We suggest the hypothesis that when FA(18:2) is (too) abundant in bees, it replaces FA(18:3), having deleterious effects. This is similar to the hypothesis suggested for mammals, that FA(18:2) replaces FA(22:6) and other PUFAs in phospholipids when intake of FA(18:2) dominates over the PUFAs (Lassek & Gaulin, 2014; Taha et al., 2014).

There is also evidence that the supply of unsaturated fatty acids to the thoracic muscle is important in flight in orchid bees (Rodríguez et al., 2018; Rodríguez et al., 2015). Specifically, the thorax temperature increased with increasing body mass of the bee (Rodríguez et al., 2018). This is consistent with the lower surface-area-to-volume-ratio of larger animals leading to slower heat loss. However, this warmer bodily temperature presents a problem in that the phase behaviour of lipids, and thus the physical behaviour of lipid membranes, is temperature dependent. Thus for a thoracic temperature of ∼ 42 °C, different lipids are optimum for membranes to those for a temperature of 32 °C. It is reported that in smaller (cooler) bees the membranes are composed of FA(16:0) and FA(18:2) whereas in larger, warmer bees the dominant FA in phospholipids is FA(18:1). The phospholipid fraction of adult worker and drone honey bees is typically dominated by phosphatidylcholine (Martin et al., 2019), a lipid whose biophysical behaviour has been studied in great detail. Biophysical studies show that the melting temperature of PC(16:0/18:2) is above that of PC(18:1/18:1) (Koynova & Caffrey, 1998), suggesting that the phospholipidome of the flight muscle of larger, warmer bees is dominated by a more fluid lipid profile than that of smaller, cooler ones. Taken together, the studies about PUFA-containing lipids in bees suggest that bees rely on polyunsaturated FAs for maintaining membrane function in several, probably all, tissues.

The physical behaviour of lipids may also be being exploited for defence. Phospholipase A_2_ (PLA_2_) is a lipase that catalyses the hydrolysis of the fatty acid residue on the *sn*-2 position of the glyceryl moiety of phospholipids to form a *lyso-*phospholipid and a fatty acid. The presence of both fatty acids and *lyso-*lipids is dangerous in membranes in vivo because they can drive the formation of non-bilayer lipid phases, thus weakening membranes (Lu et al., 1997; Slater et al., 1989; Templer et al., 1998). The PLA_2_ found in bee venom forms a considerable part of it, > 10% dry mass (Kuchler et al., 1989). It has therefore been suggested that this enzyme forms an important part of the defensive effects of a bee sting, through hydrolysis of a range of phospholipids found across the animal kingdom (Six & Dennis, 2000).

As well as physical effects of lipids on phase behaviour, they can also assist in protein binding. There are at least two isoforms of a phosphatidylethanolamine-binding protein. This protein plays a pivotal role in moulting and immunity. Indeed, the study reported on this topic concluded that the abundance of these two isoforms could be related to regulation of development in bumble bees (Dong et al., 2017). This is remarkable because it suggests that phosphatidylethanolamine (PE), a lipid that is the second most abundant in mammals (van Meer & de Kroon, 2011) and the most abundant in several bacteria (Furse et al., 2017; Furse et al., 2015b), may be a powerful signalling molecule in bees. This suggests that as well as being a straightforward membrane lipid in bees of all castes (Hu et al., 2021; Martin et al., 2019; Singh & Singh, 1996), PE is a signalling lipid. This is remarkable because the way the PE fraction is typically controlled is through more basic mechanisms. However, if it is a signalling molecule in bees, PE’s distribution may be controlled quite differently in bees than many other organisms in order that it can act as such.

The evidence collected to date about phospholipid metabolism in bees suggests a strong presence of these lipids in bees with many of the same general roles as in other terrestrial organisms. However, our understanding of phospholipid metabolism in bees is relatively weak at present and so the detailed metabolic networks that are known for mammals (reviews, Vance & Choy, 1979; Vance & Vance, 2004; Vanhaesebroeck et al., 2001) cannot yet be constructed for bees. This means the scope of endogenous lipid biosynthesis of bees is unknown, as are the mechanisms by which lipid metabolism is controlled and adapts to stress.

### Glycerides

Glycerides comprise a glyceryl moiety with one, two or three fatty acid residues. The principal member of this class of molecule in nature is the triglyceride, though there is evidence for the presence of and roles for monoglycerides and diglycerides in vivo. Triglycerides are important in nature as they provide the densest and most efficient store of energy in biological systems, carrying around 9 kcal/g against around 4 kcal/g for either carbohydrate or protein (example TG shown in Fig. 1B). This is reflected in mammals such as humans who typically store 1–2 g glucose per kilogramme bodyweight against 100–200 g/Kg of fat (men) or 250–350 g/Kg (women). Insects have specialised cells for storing fats called trophocytes (similar to adipocytes). These are found in the fat body alongside oenocytes (similar to hepatocytes). This organ has been recognised as important in insects as a store and supply of energy (review, Arrese & Soulages, 2010). The presence of a fatbody suggests that bees are reliant upon fats as a supply of energy but have not been exposed to the same selection pressures as organisms with several distinct adipose depots, e.g., humans. Furthermore, in order for bees to fly, weight should be kept to a minimum. Oxidation of FAs has not been found to occur in the CNS of honey bees (Wegener, 1983). However, lipoprotein lipase activity has been measured in the flight muscles of bees (Crabtree & Newsholme, 1972), suggesting that fats are used as fuel for flight, though at least one report suggests that the activity of this protein is not as strong as in other insects (Crabtree & Newsholme, 1972). This might be explained by a mechanism that appears in mammals. Mammals have intermuscular adipocytes that provide a ready supply of fat for muscular activity (Vettor et al., 2009), lessening the need for high lipoprotein lipase activity. However, some bees use fats during the winter when carbohydrate is not available, *e.g. Osmia lignaria* (Bosch et al., 2010, review, Sinclair & Marshall, 2018).

Triglycerides in the fat body of males from 11 bumblebee species were studied by Kofroňová et al. (2009). The composition was found to differ between species. Two hypotheses for the functional importance of differences between species were suggested based on the findings in this exploratory study. First, higher proportions of unsaturated fatty acids in membranes could be an adaptation to colder climate. However, the storage of shorter and unsaturated FAs in TGs is at odds with the physical need for them to be in PLs. Furthermore, maintaining better fluidity under cold conditions requires the presence of more fluid FA residues in phospholipids. This pattern has been observed in *Drosphila*, where TGs were depleted of unsaturated FAs under colder conditions, presumably to supply PLs (Enriquez & Colinet, 2019; Ohtsu et al., 1993). Second, particular fatty acids could be stored in triglycerides as precursors for the marking pheromones of male bumblebees. For example, three fatty acids found in triglycerides of several species share structural similarity with known male pheromones. Support for the latter hypothesis was later found in comparing male marking pheromones and fat body triglycerides in *Bombus ruderatus*, *B. bohemicus*, and *B. campestris* (Kofroňová et al., 2014). If true, this would be remarkable because it would mean that TGs are being used to store low-abundance FAs that are precursors for signalling molecules in bees. This would contrast with mammals in which polyunsaturated FAs tend to be stored in PLs (Furse & de Kroon 2015) though is consistent with compartmentalisation of adipose by TG structure in mammals (Strandberg et al., 2008). It is not clear that TGs provide the best medium for storage and recall of the appropriate FAs however only small amounts of pheromones are required so only modest release from stores is required.

Several species of solitary bee produce triglycerides to supply energy to larva. Triglycerides are secreted by the Dufour’s gland in the solitary bee *Anthophora abrupta.* The triglycerides are secreted as nest cell lining and into the pollen provision, where they undergo partial lipolysis to produce diglycerides with a lower melting point that serve as food source for the larva (Norden et al., 1980). Additionally *Anthophora* and leafcutter bee (*Megachile*) species have been found to provision their nest chamber with triglycerides from the Dufour’s gland (Cane & Carlson, 1984; (Williams et al., 1986), suggesting that provision of TGs may be commonplace in solitary bees. The main triglyceride-lipase from the insect fat body is an active phospholipase A_1_, that releases DG from TG (Arrese et al., 2006). This would be dangerous in mammals as DG is a potent molecular signal, activating protein kinase C. Presumably the spatial distribution of DGs is well controlled in bees, presumably restricted to Darfour’s gland, to avoid the same effect in bees.

The principal role for TGs as a means for storing and distributing energy in bees suggests that they have a similar general role in insects as in mammals. It also indicates that TGs may be less important for the CNS in both classes. However, it is not clear whether the supply of FAs is controlled in the same way in bees as it is in other animal clades (reviews of FA metabolism in bees, Arrese et al., 2001; Canavoso et al., 2001; Majerowicz & Gondim, 2013). For example, which FAs are produced or modified endogenously, and where, by bees has not yet been fully mapped. There is considerable evidence for production of saturated fatty acids such as steric acid in the wax glands (Blomquist et al., 1980), however this fatty acid is of very low abundance in the phospholipid or triglyceride fractions, suggesting it is not used to supply energy. Further work in this area might also be useful for understanding the distinctions between castes to uncover caste-specific differences in FA metabolism in bees (Pereboom, 2001).

In general, the evidence on the metabolism of glycerides in bees suggests that they may be as central to the supply and distribution of energy as they are in mammals (Florant, 2015; Strandberg et al., 2008), fish (Henderson, 1996) and birds (Araújo et al., 2019; (Guglielmo, 2010). Importantly, glyceride biosynthesis relies entirely on FA metabolism. Current understanding of how the FAs that are used to produce TGs are made and handled in bees is discussed next.

### Fatty acids

Fatty acids are a key component of biological systems, being used to produce triglycerides (energy storage and distribution) and phospholipids (membrane components, signalling molecules), and to lipidate macromolecules. In bees they are also used as external structural molecules (wax). The existence of FAs is believed to be a key part in the emergence of the cellular structure we are familiar with (Gaylor et al., 2021; Todd et al., 2022), with archaeal, eukaryotic and prokarytic life all relying upon the ability to produce carbon chains (Cronan & Thomas, 2009; Guillou et al., 2010; Yang et al., 2015). Fatty acids, FAs, consist of a chain of carbon atoms with a carboxylic acid group at one terminus (example FA and FA residues in lipids, Fig. 3). Chains of up to and including six carbons are miscible in water and thus acetic, butanoic and hexanoic acids do not really conform to the physical properties of typical fatty acids. FAs found in mammals often comprise olefin bonds, whereas in Gram-positive bacteria FAs can be branched and under special circumstances Gram-negative bacteria produce FAs comprising cyclopropyl groups. Hydroxyl groups are installed on FAs in mammals, but this is associated with the biosynthesis of prostaglandins rather than FAs themselves. To date, FA profiling in bees has identified mainly C_16_ and C_18_ FAs, with up to three double bonds (Wegener et al., 2018), which is similar to a simplified mammalian FA composition. There is considerable variety in FA composition amongst species of bee (Giri et al., 2018), however it is not clear whether this can be ascribed only to diet. In several bees the FA composition reflects the FAs found in bee bread (Martin et al., 2019; Martin et al., 2022) and dietary pollen (DeGrandi-Hoffman et al., 2018; Kaplan et al., 2016; Manning, 2001). There is some evidence that wasps have selectivity over FA uptake, with long chain polyunsaturated FAs in the diet not found in quantity in the body (Giri et al., 2018). These straightforward FAs are used to produce phospholipids and triglycerides and thus perform the typical functions of fats and lipids in animals and plants. The evidence of transcriptional control of FA consumption during the whole lifetime of the honey bee (Ament et al., 2011) suggests that FAs represent a valuable resource to bees. The FA profile of the lipidome of bees is more select than that in the pollen fed to them (Martin et al., 2019; Martin et al., 2022) suggesting careful filtering/control of FAs *in apia*. However, where fatty acids are supplied as triglycerides, bees can become obese (Stabler et al., 2021), suggesting only partial regulation of FA intake. This contrasts with regulation of protein intake, that is more precise (Stabler et al., 2021), and raises questions about how fat intake is regulated in oil-collecting bees.

FA metabolism falls into three parts: biosynthesis, use (including storage) and degradation. All of these have been studied in bees. Reports of studies of endogenous synthesis of FAs in bees (*de novo* lipogenesis, DNL) show that bees have the enzymes needed to produce simple FAs *de novo* (Helmkampf et al., 2014; Teerawanichpan et al., 2010), in contrast to related insects such as parasitoid wasps that are known not to be capable of DNL at all (Snart et al., 2018). However, linoleic acid is essential for development of honey bees (Arien et al., 2018; Arien et al., 2015) but it appears cannot be made *de novo* in this organism. This is in contrast to several other species of insect that can produce all of the linoleic acid they need (M.Th. Beenakkers et al., 1985).

It is therefore assumed that bees fed on a diet of sugar water in laboratories can survive because they produce FAs from the sugars they consume. However, in free-flying bees, the size of the fatbody has been found to be linked to foraging behaviour (Toth et al., 2005; Toth & Robinson, 2005), suggesting that energy expenditure is a key player in shaping the profile of TG and of FAs in bees.

**Fig. 4:**
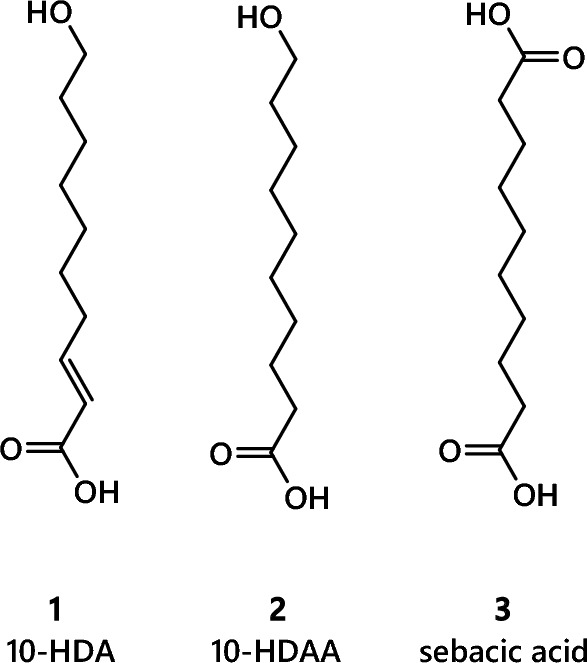
Molecular structures of FAs found in abundance in Royal Jelly made de novo in *Apis mellifera*

The limits of endogenous FA biosynthesis for lipid, rather than wax, production in bees therefore represents a considerable gap in our knowledge. Part of the reason for this is that research on FA metabolism in bees has focused on the biosynthesis and uses of a different FA altogether, and one that may be unique to bees. Queen bee acid, 10-hydroxydecenoic acid (10-HDA, **1**), comprises a trans-double bond and a terminal hydroxyl group on a ten-carbon chain and is typically found in Royal Jelly (RJ) with the closely related 10-hydroxydecanoic acid (10-HDAA, **2**) and sebacic acid (**3**), Fig. 4 (Dixon & Shuel, 1978; Spannhoff et al., 2011; Terada et al., 2011). Most studies to date have focused on **1** and **2**, with a considerable body of evidence showing that both are consistently present in RJ wherever sampled (Ferioli et al., 2014; Howe et al., 1985; Isidorov et al., 2012; Lercker et al., 1981; Lercker et al., 1982; Wang et al., 2016; Wytrychowski et al., 2013; Xu & Gao, 2013; Zheng et al., 2011). This has also led to the generation of standard methods for investigating RJ, allowing samples collected in different places at different times to be compared more easily (Hu et al., 2019).

The lipid fraction forms around 5–15% of the mass of RJ. **1** represents 50% of the lipid fraction, with 10-HDAA, **2**, typically forming around 25% of the lipid fraction (Isidorov et al., 2012; Lercker et al., 1981; Lercker et al., 1982). The remaining portion of the lipid fraction (∼ 25%) consists of sebacic acid and several shorter-chain fatty acids and hydroxylated FAs (Ferioli et al., 2014). The presence of so much of **1** during the early development of bees has raised questions about the metabolic or developmental function of this compound. There is evidence for activity as a histidine deacetylase inhibitor and TRPA1 activator (Spannhoff et al., 2011; Terada et al., 2011), and a general role in development (Dixon & Shuel, 1978; Kinoshita & Shuel, 1975; Šedivá et al., 2018). However, the range of effects that **1** and **2** produce or contribute to is unclear, as is how they are controlled.

Intake of **1** and **2** throughout the larval stages correlates with development of queens, with workers emerging from larvae fed stored pollen from around a third of the way through development. This is around the point that drone larvae are provisioned stored pollen, too. This change in food is known to represent a change in the intake of **1** and **2** by the larva, leading to suggestions that **1** and **2** have role(s) as signalling molecules. Evidence for both inhibitory and activation effects of **1** on enzymes (Spannhoff et al., 2011; Terada et al., 2011) suggests that like many other lipids, it may perform a variety of functions simultaneously. This necessitates precise control by the organism and makes it difficult to either pinpoint or fully characterise the role of the lipid in vivo.

However, although there is evidence for signalling roles, **1** and **2** are several orders of magnitude much more abundant in RJ than other lipid signalling molecules found elsewhere in nature, such as inositides or lipokines. This means that a signalling role would have to be controlled in a unique manner, i.e., constructed differently to all known lipid signalling systems. This suggests that destinations of **1** and **2** that are stoichiometric may also be important. There are several features that could contribute to onward metabolism of these compounds. **1** and **2** have a terminal (ω-1) hydroxyl group, meaning they can be phosphorylated or form dimers or polymers (Noda et al., 2005)^133^. The terminal hydroxyl also means that these compounds are similar to aliphatic alcohols that are also made endogenously in *Apis* spp. (Teerawanichpan et al., 2010), for example for producing wax. A stoichiometric role for these FAs is consistent with our understanding of lipid metabolism in mammals and insects that shows that it is unlikely that so much carbon would be discarded despite its energetic value or possible use as a structural molecule. However, it is not known where the majority of **1** and **2** go. There is evidence of the expression of both elongases and desaturases during development (Falcón et al., 2014), suggesting the hypothesis that **1** and **2** are elongated. This is also co-temporal with formation of the exoskeleton, suggesting that there is demand for carbon for non-cellular structural roles.

Elongases are also crucial in the biosynthesis of beeswax. Beeswax consists of classical waxes about 85% (Serra Bonvehi & Orantes Bermejo, 2012; Tinto et al., 2017; Tulloch, 1970; Tulloch, 1980) and decarboxylated chains (hydrocarbons with odd numbers of carbon atoms). Genetics and transcriptomics studies show that wax production is probably driven exclusively by *de novo* lipogenesis, through the expression of appropriate genes (Teerawanichpan et al., 2010) coupled with appropriate genes for synthesis from sugars (Glimcher & Lee, 2009). In a study carried out using hives in South Africa, the composition of wax from newly-emerged bees was found to be dominated by FA(16:0) and FA(24:0), suggesting that long, saturated FAs can be produced *in apia*. Importantly, the profile changed after emergence, with FA(12:0) was detected from day 12 and rose at the expense of FA(16:0) and FA(24:0) to around 29% by day 21 (Hepburn et al., 1991). The composition of beeswax also varies by geographical location (Tinto et al., 2017). This is doubly remarkable as it shows that the range of FAs in wax is wider and less unsaturated than in those in lipids. These studies therefore suggest the hypotheses that endogenous lipid biosynthesis is shaped by climatic conditions, dietary availability of FAs (that are elongated) and/or bee age as well as external chemical stressors. Biosynthesis of carbon chains for wax that is sensitive to local temperature would be consistent with our understanding of the biophysics of lipid systems; the wax used to make a honeycomb must be neither too brittle nor too soft to be viable in the temperature range in which the colony lives. A major factor in lipid metabolism in *Apis* spp. is the biosynthesis of wax.

These studies show that bees possess a well-developed and specific FA biosynthesis and put FAs to a variety of uses. This includes oxidation, i.e. the use of FAs as fuel. The existence of a well-developed FA metabolism and a reliance on the energy and structural roles of FAs raises the possibility that the control of the system could be modulated by external factors such as enzyme inhibitors. Exposure of *Bombus terrestris* workers bees to the neonicotinoid insecticide imidacloprid was found to suppress the mevalonate and fatty acid synthesis pathways (Erban et al., 2019), suggesting that this pesticide may disrupt lipid metabolism. This suggests that not only is FA metabolism well developed in bees but it is also sensitive to chemical stresses. However, such stressors are not necessarily intended as toxins. Pantothenic acid, vitamin B_5_, also has what appears to be a damaging effect on lipid metabolism in bees (Hu et al., 2022), in contrast to its essential role in humans. Perhaps more surprisingly, FA(18:1) appears to shorten longevity in honey bees when taken at > 2% of dietary intake (Manning et al., 2007). This is despite evidence for a role in enhancing learning and survival (Muth et al., 2018). FA(18:2n-6), α-linolenic acid also has an important role in honey bee colony development (Ma et al., 2015) and learning (Arien et al., 2018; Arien et al., 2015), and is associated with longer lifespans at > 6% of dietary intake in honey bees.

The evidence for DNL of both straightforward FAs and unique ones, and a reliance on dietary intake for others, suggests that lipid metabolism in bees shares characteristics with that in other animal clades. As well as *de novo* lipogenesis, bees are also known to be capable of some manipulations of FAs such as desaturation (Helmkampf et al., 2014), and adaptable production of wax (Tinto et al., 2017). These effects suggest that bees have a carefully controlled but sensitive FA metabolism, with some absolute requirements from dietary intake. However, the limits of the system are unclear, as are its ability to resist chemical and environmental stresses.

### Future perspectives

The current evidence suggests the scope of lipid metabolism is wide in bees. The evidence for endogenous synthesis of both short and long FAs (C_12_ and C_40_ + respectively) (Hepburn et al., 1991; Serra Bonvehi & Orantes Bermejo, 2012; Tinto et al., 2017; Tulloch, 1970; Tulloch, 1980), the expression of desaturases and elongases, existence of unique hydroxylated FAs, and the abundance of mid-length FAs (C_16–18_) in the phospholipid fraction (Martin et al., 2019) suggests that bees have a lipid metabolism comparable to or more diverse than mammals and plants in overall range and complexity. The evidence that the FA and sterol composition of beeswax, phospholipids and royal jelly all differ considerably leads us to suggest that the control of lipid metabolism in bees is both precise and different to that in mammals. However, unlike mammals, *de novo* lipogenesis is poorly understood in bees. This highlights an important area for further study. The sterol metabolism of bees is better understood, however significant questions about the onward metabolism of sterols remain. The biggest of these is probably why the most abundant sterol (24-methylenecholesterol) appears to be both metabolically isolated from other sterols and an absolute dietary requirement. It is currently not known whether this sterol is glycosylated in vivo. Important outstanding questions remain about the origin and fate of ten-carbon FAs in glandular secretions fed to larvae (e.g. royal jelly). Specifically, it would be exciting to discover that these compounds represent a form of lipid signalling that is distinct from those found in mammals, such as PIP_2_ or PPAR. Third is phospholipid metabolism, which is currently poorly explored in bees. Fourth is understanding the apparent differences in lipid metabolism between bee species as these distinctions may be the result of the evolution of host plant specialisation of bees. Perhaps the most important in the context of a changing climate is whether the foraging requirements of bees are met in anthropogenically modified landscapes.

The role of lipid metabolism in metabolic disease is beginning to emerge in mice and humans, and has been shown to be an important factor for longevity of individuals and the metabolic health of succeeding generations. Evidence for a relationship between dietary intake and longevity has been observed in other insects (Piper & Partridge, 2007; Stefana et al., 2017) and thus represents a possible threat to long-term bee health and colony viability. However comparatively little is known about the direct relationship between dietary intake and metabolic health or development in bees. This therefore suggests that fundamental questions about lipid metabolism are relevant to furthering our knowledge of development and longevity *in apia.*

All the gaps in our knowledge represent limits in our ability to secure the long-term health of bees for agriculture and thus represent areas for urgent study. Collection of nutritional data, requiring multi-omics approaches, could then be used for wholistic as well as specific analyses of nutrition in pollinators. Analysis tools such as the Geometric Framework for Nutrition (Raubenheimer & Simpson, 2016; Simpson et al., 2017; Solon-Biet et al., 2018) and Traffic Analysis (Furse et al., 2022b; Furse et al., 2021) can then be used to understand nutritional needs and future questions. This could be particularly important for solitary bees that are less well studied even that other bees. Intriguingly, the evidence for careful control of a broad and flexible lipid metabolism in bees, coupled with the size of the organism being large enough for lipidomics data collection but small enough to be convenient for laboratory study, suggests it may provide a very useful model of lipid metabolism as well as for its own needs. It may even be capable of answering questions that mammalian and smaller insect models cannot. However, the urgent questions around sterol and lipid metabolism in bees rest on the lack of plentiful supply of appropriate nutrients, driven by a changing landscape and the continued need for biotic pollination for terrestrial life to remain viable.

## Abbreviations

24MC: 24-Methylenecholesterol
δ5: Delta-5, a sterol with a double bond between carbons 5 and 6 in the B ring
δ7: Delta-7, a sterol with a double bond between carbons 7 and 8 in the B ring
DNL: *de novo* lipogenesis
FA: Fatty acid
HDA: 10-Hydroxydec-2-enoic acid
HDAA: 10-Hydroxydecanoic acid
LPC: *lyso-*Phosphatidylcholine
PC: Phosphatidylcholine
PE: Phosphatidylethanolamine
PI: Phosphatidylinositol
PLA_2_: Phospholipase A_2_
PS: Phosphatidylserine
PUFA: Polyunsaturated fatty acid
SM: Sphingomyelins
